# Temporal Evolution of Perihematomal Blood-Brain Barrier Compromise and Edema Growth After Intracerebral Hemorrhage

**DOI:** 10.1007/s00062-023-01285-z

**Published:** 2023-04-25

**Authors:** Dan Yang, Xin Wang, Xue Zhang, Huachen Zhu, Shengjun Sun, Ravikiran Mane, Xingquan Zhao, Jian Zhou

**Affiliations:** 1 Department of Radiology, Beijing Tiantan Hospital, Capital Medical University, No.119, South Fourth Ring West Road, Fengtai District, 100070 Beijing, China; 2 Department of Neurology, Beijing Tiantan Hospital, Capital Medical University, Beijing, China; 3 Department of Neuroradiology, Beijing Neurosurgical Institute, Beijing Tiantan Hospital, Capital Medical University, Beijing, China; 4 China National Clinical Research Center-Hanalytics Artificial Intelligence Research Centre for Neurological Disorders, Beijing, China

**Keywords:** Permeability-surface area product, Secondary brain injury, Functional outcome, Computed tomography perfusion, Stroke

## Abstract

**Purpose:**

The aim of this study was to investigate the temporal evolution of perihematomal blood-brain barrier (BBB) compromise and edema growth and to determine the role of BBB compromise in edema growth.

**Methods:**

Spontaneous intracerebral hemorrhage patients who underwent computed tomography perfusion (CTP) were divided into five groups according to the time interval from symptom onset to CTP examination. Permeability-surface area product (PS) maps were generated using CTP source images. Ipsilateral and contralateral mean PS values were computed in the perihematomal and contralateral mirror regions. The relative PS (rPS) value was calculated as a ratio of ipsilateral to contralateral PS value. Hematoma and perihematomal edema volume were determined on non-contrast CT images.

**Results:**

In the total of 101 intracerebral hemorrhage patients, the ipsilateral mean PS value was significantly higher than that in contralateral region (z = −8.284, *p* < 0.001). The perihematomal BBB permeability showed a course of dynamic changes including an increase in the hyperacute and acute phases, a decrease in the early subacute phase and a second increase in the late subacute phase and chronic phase. Perihematomal edema increased gradually until the late subacute phase and then slightly increased. There was a relationship between rPS value and edema volume (β = 0.254, *p* = 0.006).

**Conclusion:**

The perihematomal BBB permeability is dynamic changes, and edema growth is gradually increased in patients following intracerebral hemorrhage. BBB compromise plays an essential role in edema growth. The quantitative assessment of BBB compromise may provide valuable information in therapeutic interventions of intracerebral hemorrhage patients.

**Supplementary Information:**

The online version of this article (10.1007/s00062-023-01285-z) contains supplementary material, which is available to authorized users.

## Introduction

Spontaneous intracerebral hemorrhage is a common subtype of stroke with high morbidity and mortality [1]. The unfavorable outcomes of intracerebral hemorrhage patients are commonly attributed to secondary brain injuries that contribute to neurological deterioration after symptom onset apart from the primary injury [2, 3]. Perihematomal edema growth is one of the crucial markers of secondary brain injury which leads to poor clinical prognosis [2, 4, 5]. As a structural foundation of edema formation, the integrity of the blood-brain barrier (BBB) is disrupted after hemorrhage, then promotes fast edema growth especially in vasogenic edema [6–8].

The BBB is composed of capillary endothelial cells, basal lamina, tight junctions and end-feet of astrocytes, and plays a key role in maintaining the homeostasis of the neural microenvironment [9–11]. The quantitative assessment of BBB compromise may provide valuable information in clinical intervention and prognostic evaluation for cerebrovascular diseases [12, 13].

The elevated BBB permeability has been observed in the region around the hematoma at different time phases in intracerebral hemorrhage patients using computed tomographic perfusion (CTP) images, and the extent of perihematomal BBB compromise has been evaluated by permeability-surface area product (PS) value [14, 15]. Some experimental longitudinal studies have revealed the dynamic alterations of BBB permeability in intracerebral hemorrhage progression [16, 17], and BBB injury has been thought to be a crucial factor in edema formation and growth [18, 19]. Several clinical studies have also shown the increased perihematomal BBB permeability within 24–72 h after hemorrhage [14, 15]; however, the temporal evolution of BBB compromise and its effect on edema growth in intracerebral hemorrhage patients are still unclear. Therefore, further research is needed to get a comprehensive understanding of BBB compromise and edema growth after hemorrhage. We hypothesized that perihematomal BBB compromise may promote edema growth and lead to poor functional outcomes in intracerebral hemorrhage patients.

In this study, we aimed to investigate the temporal evolution of perihematomal BBB compromise and edema growth after intracerebral hemorrhage as well as the related factors using CTP imaging, to provide valuable information for future therapeutic interventions aimed at alleviating cerebral edema in patients with intracerebral hemorrhage.

## Material and Methods

### Patients

Patients with spontaneous symptomatic intracerebral hemorrhage admitted to the emergency, outpatient, and inpatient departments of Beijing Tiantan Hospital from November 2019 to October 2021 were prospectively enrolled in this study according to the following inclusion criteria: (1) symptomatic intracerebral hemorrhage patients confirmed by baseline non-contrast CT scans, (2) age ≥ 18 years, (3) spontaneous intracerebral hemorrhage, (4) availability of CTP examination and (5) known time interval from symptom onset to CTP examination. Exclusion criteria for this study were as follows: (1) infratentorial intracerebral hemorrhage, (2) secondary hemorrhage including trauma, tumor, underlying aneurysm, vascular malformation, hemorrhagic venous infarct, hemorrhagic transformation of ischemic stroke, vasculitis, illicit drug usage, septicemia and other rarer causes, (3) lack of CTP scans due to known contradictions to CTP, (4) neurosurgical intervention prior to CTP, (5) lack of informed consent, (6) poor quality CTP/CT scans and (7) lack of complete clinical information. Additionally, a few severely ill intracerebral hemorrhage patients with high National Institutes of Health stroke scale (NIHSS) score or low Glasgow Coma Scale (GCS) score were excluded from the study due to the possibility of allergic reactions to the CTP contrast medium.

Depending on the time from intracerebral hemorrhage onset to CTP examination, intracerebral hemorrhage patients were divided into five subgroups: hyperacute (< 1 day), acute (1–3 days), early subacute (3–7 days), late subacute (7–14 days) and chronic (> 14 days). We collected modified Rankin Scale (mRS) scores at 90 days after the ictus and patients were divided into the good prognosis group (mRS score ≤ 2) and the poor prognosis group (mRS score ≥ 3). Stroke physicians collected detailed information about patients by using standard questionnaires at presentation. Demographic information and vascular risk factors such as a history of hypertension, diabetes, previous cerebral vascular event, and location and volume of hematoma were systematically recorded.

### Imaging Acquisition

Intracerebral hemorrhage patients underwent non-contrast CT at admission on a CT scanner (Revolution CT, GE Healthcare, Chicago, IL, USA) with 120 kVp, 100 mA, 5 mm section thickness, 5 mm intersection space, 512 × 512 image matrix, 1 s gantry rotation time and helical pitch of 0.984:1. Following non-contrast CT, a two-phase CTP examination was performed with a 140 mm displayed field of view from the vertex of the skull to the foramen magnum level. In the first phase of CTP examination, images were collected in cine mode for 45 s and were reconstructed at 0.5 s interval. The second phase involved a 90‑s image acquisition at a 15‑s interval after the first phase. Scanning parameters were 80 kVp, 100 mA, 8 × 5 mm collimation, 5 mm section thickness, 10 mm intersection space, 512 × 512 image matrix, and a gantry speed of 1 s per rotation. For contrast, 40 mL of iohexol (350 mg/mL, Omnipaque, GE Healthcare) was injected into an antecubital vein by a power injector at a rate of 4 mL per second followed by a saline flush of 20 mL. The CTP scan was initiated after a delay of 5 s from contrast injection.

### Image Postprocessing

**Fig. 1:**
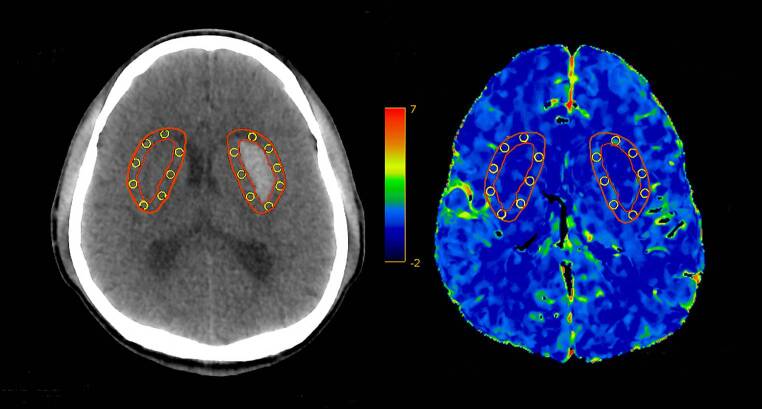
Graphical illustration of the regions of interest (ROIs) drawn on computed tomography perfusion baseline image (**a**) and corresponding permeability-surface area product map (**b**). The region between two red lines defines a 10mm zone surrounding the hematoma. *Yellow circles* define ROIs drawn in the perihematomal region

Postprocessing of raw CTP source images was performed on CT Perfusion 4D software of a standard Advantage Workstation (AW 4.7, GE Healthcare). The software performed head motion correction and generated mathematical descriptions of time-density curves for each brain voxel. Arterial input and venous output were derived from the ipsilateral anterior cerebral artery and the superior sagittal sinus. The parametric maps PS were calculated by the same software using a deconvolution algorithm. The PS value is a measure used to assess the rate of contrast leakage from the intravascular to extravascular space through a disrupted BBB. Regions of interest (ROIs) were drawn using planimetric techniques on raw CTP images and were transferred to corresponding PS maps. At the image slice with the maximum hematoma area, 8 approximately equidistant 80mm^2^ round ROIs were marked within the 1 cm boundary region outside the hematoma. Contralateral mirror ROIs were constructed by reflecting the ROIs around hematoma across the brain midline (Fig. 1). Mean PS values were calculated by averaging the PS values in 8 ROIs in each brain hemisphere. To minimize the impact of patient level heterogeneity, relative PS (rPS) value was calculated as a ratio of ipsilateral to contralateral mean PS. Large blood vessels, ventricles, image artifacts, bones, and calcifications were avoided while drawing the ROIs to ensure more accurate and repeatable results. This CTP post-processing was independently completed by two experienced radiologists.

### Statistical Analysis

The data analyses were performed in the SPSS software (version 23.0; IBM, Armonk, NY, USA). Quantitative variable results were expressed as mean and standard deviation (SD) or median and interquartile range (IQR), and categorical finding results were expressed as proportions. The ipsilateral and contralateral PS values were compared using Wilcoxon signed rank test. Mann-Whitney U-test was used to compare rPS values between men and women, hemorrhages in lobar and deep locations, patients with and without hypertension, and patients with and without diabetes. The relationships between age, hematoma volume, edema volume and rPS value were assessed using linear regression. Univariate analyses were performed to identify the factors having a significant relationship with mRS score at 90 days after intracerebral hemorrhage. Univariate analysis included a χ^2^-test for categorical data, and Student’s t‑test or Mann-Whitney U-test for continuous data. Significant variables in univariate analyses (*p* < 0.1) were included in a multivariate logistic regression to identify independent factors associated with functional outcomes for patients with intracerebral hemorrhage. The intraclass correlation coefficient was calculated to evaluate inter-rater repeatability of PS values. All tests of significance were 2 tailed, and a probability value < 0.05 was considered statistically significant.

## Results

### Patient Demographics

**Fig. 2:**
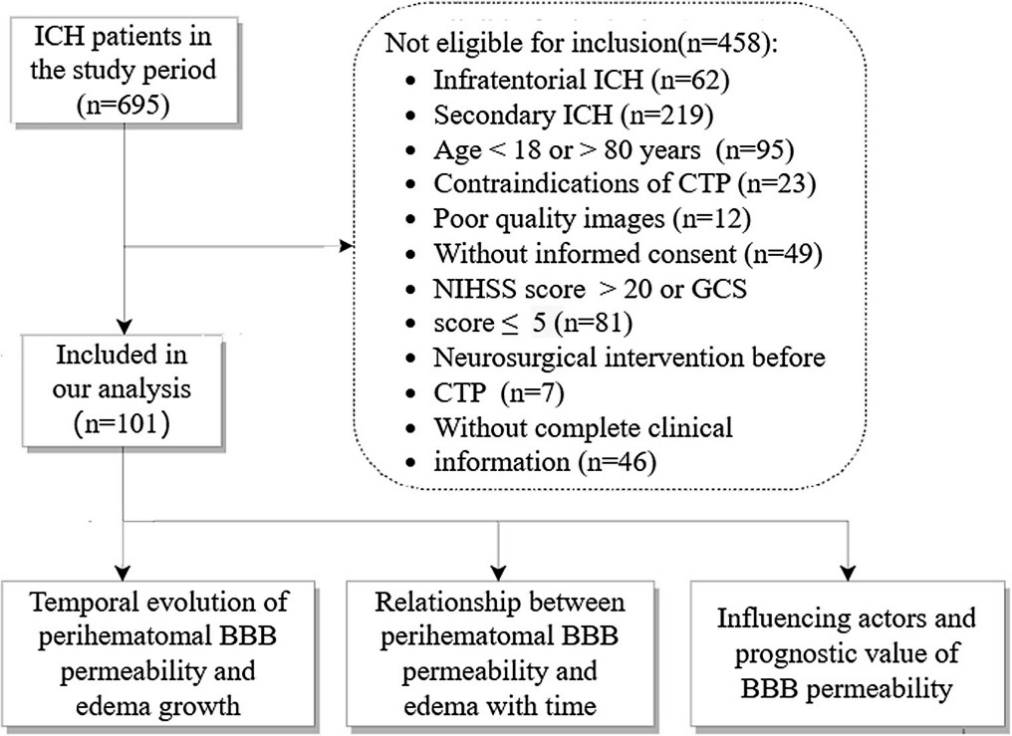
Study flow chart. *BBB* blood-brain barrier, *CTP* computed tomographic perfusion, *GCS* Glasgow Coma Scale, *ICH* intracerebral hemorrhage, *mRS* modified Rankin scale, *NIHSS* National Institutes of Health Stroke Scale, *rPS* relative BBB permeability-surface area product

A total of 101 patients with spontaneous intracerebral hemorrhage met the study inclusion criteria and fulfilled none of the exclusion criteria (Fig. 2). A strong agreement was observed between the two radiologists for BBB permeability measurements (intraclass correlation coefficient = 0.706; *p* < 0.001). The mean patient age was 52.13 years (SD: ±11.86, range: 19–77 years) and the proportion of women was 29% (29 women). Among the 101 patients, 80 had deep hemorrhages and 21 had lobar hemorrhages. Median (IQR) hematoma volume was 13.29 ml (5.20–30.11 ml). Median perihematomal edema volume was 25.60 ml (11.58–50.08 ml) when undergoing CT and CTP examination. At baseline, the median (IQR) admission NIHSS score was 8 (3–14), and the admission GCS score was 13 (10–15). The median (IQR) mRS score at 90 days was 2 (1–4).

### Measurement of the PS and rPS

CTP examinations were performed from 12 h to 23 days 3 h after intracerebral hemorrhage symptom onset. The median PS value from ipsilateral ROIs was 1.76 (0.93–3.35) ml/100 ml per min, and contralateral ROIs was 0.78 (0.43–1.23) mL/100 mL per min. The perihematomal BBB permeability was significantly increased compared to the contralateral mirror region (z = −8.284, *p* < 0.001). The demographic and clinical characteristics of the patients are summarized in Table 1.

**Table 1:** Clinical and radiographic characteristics of 101 intracerebral hemorrhage patients

*Age in years, mean* *± SD*	*52.13* *± 11.86*
*Sex (female), n (%)*	*29 (29%)*
*Clinical characteristics*	
Admission NIHSS, median (IQR)	8 (3–14)
Admission GCS, median (IQR)	13 (10–15)
90 days mRS score, median (IQR)	2 (1–4)
*Medical history*	
Hypertension, *n* (%)	87 (86%)
Diabetes, *n* (%)	16 (16%)
Hyperlipidemia, *n* (%)	48 (48%)
Ischemic stroke, *n* (%)	21 (21%)
ICH, *n* (%)	1 (2%)
*Medication history*	
Anticoagulants	13 (13%)
*Hematoma volume (mL), median (IQR)*	13.29 (5.20–30.11)
*Perihematomal edema volume (mL), median (IQR) *	25.60 (11.58–50.08)
*Hematoma location, n (%)*	
*Lobar hemorrhage (with hypertension)*	21 (21%) (15)
*Deep hemorrhage (with hypertension)*	80 (79%) (73)

*CTP* computed tomography perfusion, *GCS* Glasgow Coma Scale, *ICH* intracerebral hemorrhage, *IQR* interquartile range, *mRS* modified Rankin Scale, *NIHSS* National Institutes of Health Stroke Scale, *SD* standard deviation

### Temporal Evolution of Perihematomal BBB Permeability and Edema Growth

**Fig. 3:**
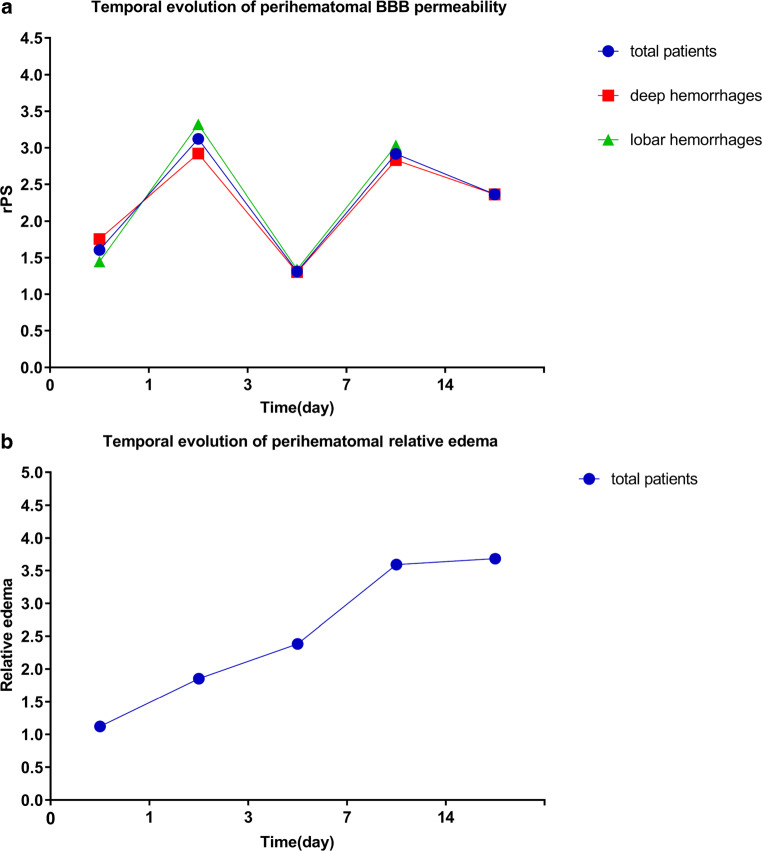
Temporal evolution of perihematomal BBB permeability (**a**) and relative perihematomal edema (**b**) in intracerebral hemorrhage patients. *rPS* relative BBB permeability-surface area product, *BBB* blood-brain barrier

Of the 101 included patients, 26 were in the hyperacute phase, 24 were in the acute phase, 22 were in the early subacute phase, 23 were in the late subacute phase, and 4 were in the chronic phase. BBB permeability showed an early increase in the hyperacute and acute phases, a decrease in the early subacute phase, and a delayed increase in the late subacute and chronic phases (Fig. 3a). The perihematomal edema volume gradually increased over time within the first 2 weeks and slightly increased thereafter (Fig. 3b). The characteristics of intracerebral hemorrhage patients grouped by different phases are summarized in Table 2.

**Table 2:** Characteristics in intracerebral hemorrhage patients grouped by time interval from intracerebral hemorrhage onset to computed tomographic perfusion examination

Characteristics	Hyperacute (< 1 day) *n* = 26	Acute (1–3 days) *n* = 24	Early subacute (3–7 days) *n* = 23	Late subacute (7–14 days) *n* = 24	Chronic (> 14 days) *n* =4
Age in years, mean ± SD	48.23 ± 14.13	54.04 ± 7.57	50.57 ± 11.35	54.42 ± 12.38	52.25 ± 9.07
Sex (female), *n* (%)	8 (31%)	8 (33%)	5 (22%)	6 (25%)	2 (50%)
Admission GCS, median (IQR)	13 (9–15)	12.5 (8.5–15)	13 (6–15)	14 (12.25–15)	15 (14.25–15)
Admission NIHSS, median (IQR)	11.5 (3–18)	11.5 (6–16.5)	7 (4-12)	6 (2.25–10.75)	7.5 (4–12.5)
Hematoma volume (mL), median (IQR)	25.15 (8.92–42.04)	19.51 (7.59–36.00)	13.90 (5.02-18.13)	6.32 (2.63–15.93)	6.03 (5.03–13.97)
Perihematomal edema volume (mL), median (IQR)	22.14 (10.77–44.52)	30.65 (16.98–63.23)	29.59 (11.45-49.45)	25.37 (10.88–47.48)	39.28 (12.73–92.32)
Relative perihematomal edema volume (mL), median (IQR)	1.12 (0.56–2.11)	1.85 (1.14–2.79)	2.38 (1.69–4.12)	3.59 (1.75‑6.12)	3.68 (2.13–14.42)
Hematoma location (deep), *n* (%)	20 (77%)	19 (79%)	18 (78%)	19 (79%)	4 (100%)
rPS (all), median (IQR)	1.61 (1.28–3.04)	3.12 (1.66–5.88)	1.31 (1.12–2.04)	2.92 (1.55–9.48)	2.37 (1.81–15.14)
rPS (deep), median (IQR)	1.76 (1.27–2.48)	2.92 (1.73–6.45)	1.30 (1.12–2.49)	2.83 (1.60–7.49)	2.37 (1.81–15.14)
rPS (lobar), median (IQR)	1.45 (1.26–9.86)	3.32 (1.55–4.39)	1.34 (1.18–1.72)	3.03 (1.26–13.86)	–

*GCS* Glasgow Coma Scale, *IQR* interquartile range, *NIHSS* National Institutes of Health Stroke Scale, *rPS* relative blood-brain barrier permeability-surface area product, *SD* standard deviation

### Relationship Between Perihematomal rPS and Edema with Time

**Fig. 4:**
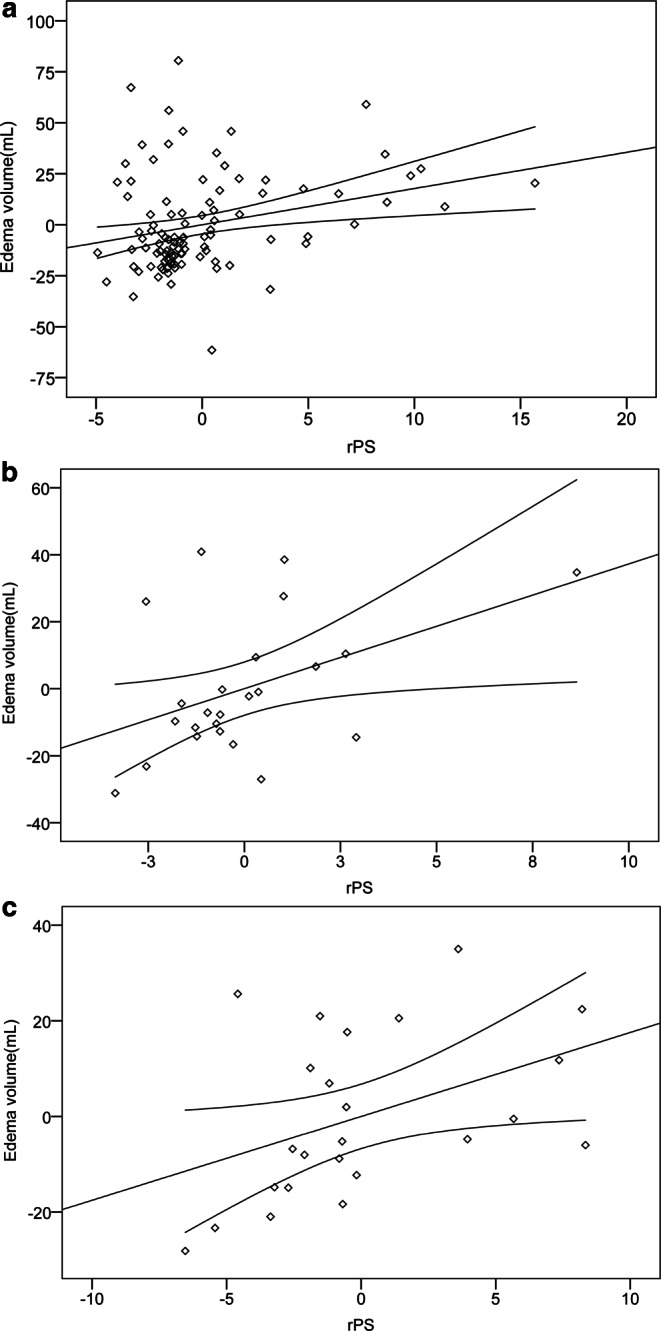
Partial regression plots showing the relationships between BBB permeability and edema volume for all patients (**a**), patients in acute phase (**b**) and patients in late subacute phase (**c**). rPS is associated positively with the perihematomal edema volume in all patients, patients in acute and late subacute phases using hematoma volume as a covariate. *BBB* blood-brain barrier, *rPS* relative BBB permeability-surface area product

Univariate linear regression analysis showed that the rPS value in the perihematomal region was significantly associated with perihematomal edema volume (β = 0.388; *p* < 0.001). The association remained significant in the multivariate regression analysis that included hematomal volume as a covariate (β = 0.254; *p* = 0.006) (Fig. 4a). In the sub-group analysis, rPS value in acute phase was associated with perihematomal edema volume (β = 0.632;* p* < 0.001), even after including hematomal volume as a covariate (β = 0.386; *p* = 0.036) (Fig. 4b). Similar relationship was observed in the late subacute phase (univariate analysis: β = 0.572, *p* = 0.0035; multivariate analysis: β = 0.332; *p* = 0.044) (Fig. 4c).

### Relationship Between rPS and Other Demographic Factors

**Fig. 5:**
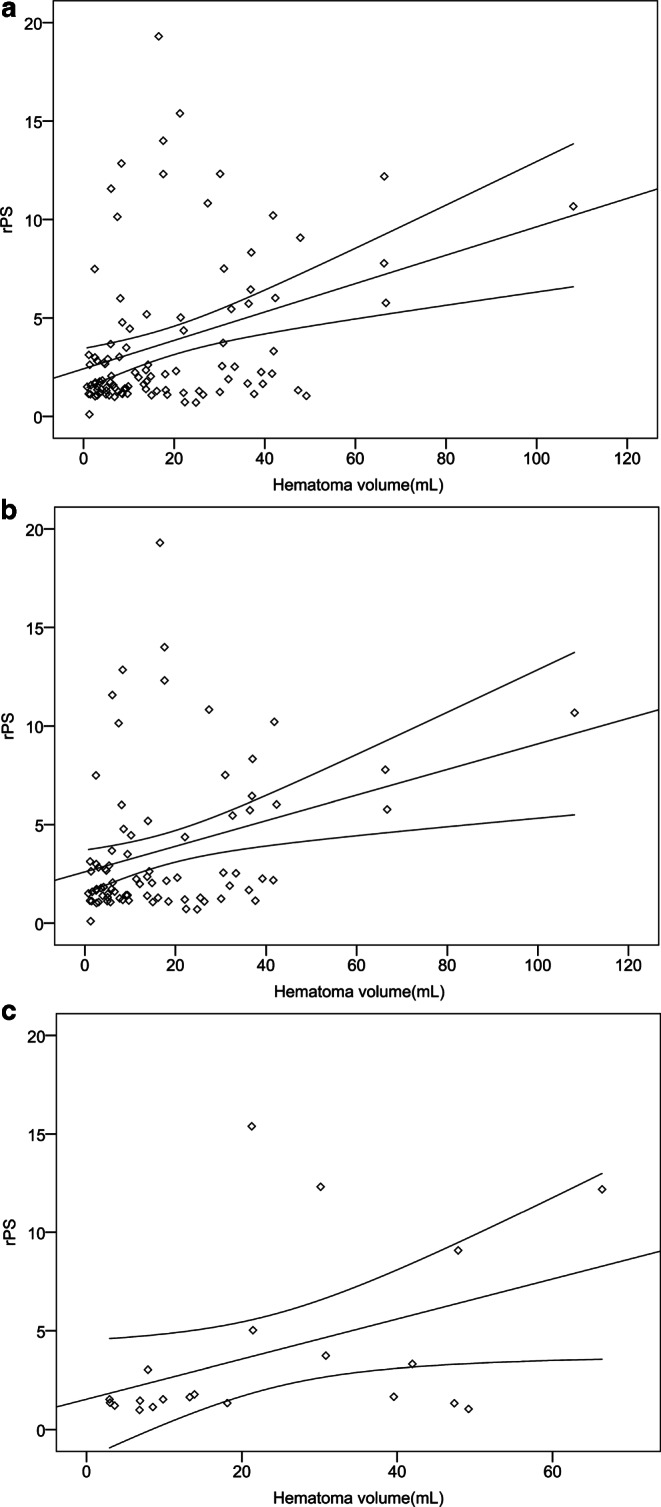
Scatter plots with linear regression coefficients and 95% confidence intervals. Perihematomal rPS is associated positively with the hematoma volume in all patients (**a**), deep hemorrhage patients (**b**) and lobar hemorrhage patients (**c**). *rPS* relative blood-brain barrier permeability-surface area product

In linear regression analysis, higher rPS values were significantly related to larger hematoma volume in all patients (β = 0.340, *p* < 0.001) (Fig. 5a) including patients with deep hemorrhage (β = 0.312, *p* = 0.005) (Fig. 5b) and patients with lobar hemorrhage (β = 0.435, *p* < 0.05) (Fig. 5c). There is no relationship between perihematomal rPS value and age (β = 0.094, *p* = 0.349). Mann-Whitney U test revealed no significant difference in perihematomal rPS value between intracerebral hemorrhage patients with deep and lobar hemorrhages (z = −0.351, *p* = 0.725), female and male patients (z = 1.419, *p* = 0.156), and with and without prior hypertension (z = 1.08, *p* = 0.280) and diabetes (z = −0.149, *p* = 0.882).

### Relationship of rPS and Other Demographic Factors with Functional Outcomes

In univariate analysis, larger hematoma volume (z = 4.841; *p* < 0.001), larger perihematomal edema volume (z = 4.031; *p* < 0.001), lower GCS score (z = −4.752; *p* < 0.001) and intraventricular hemorrhage (χ^2^ = 3.150; *p* = 0.076) were significant factors in predicting poor outcomes at 90 days. No significant difference in BBB permeability was observed between the two groups with different prognoses (z = 1.329, *p* = 0.184) (Table 3). In multivariate logistic regression model, larger hematoma volume (odds ratio = 1.054, 95% confidence interval: 1.014–1.096, *p* = 0.008) and lower GCS score (odds ratio = 0.846, 95% confidence interval: 0.745–0.961, *p* = 0.01) were independent predictors associated with poor outcomes at 90 days (Table 4).

**Table 3:** Differences in characteristics between patients with good and poor prognosis

Characteristics	Good prognosis (*n* = 56)	Poor prognosis (*n* = 45)	*p* value
Sex (female), *n* (%)	15 (27%)	14 (31%)	0.633^a^
Age in years, mean ± SD	52.91 ± 11.87	51.16 ± 11.91	0.463^b^
Hematoma location (deep), *n* (%)	45 (80%)	35 (78%)	0.751^a^
Hematoma volume in mL, median (IQR)	7.15 (3.17–17.59)	22.35 (11.36–38.11)	< 0.001^c^
Perihematomal edema volume in mL, median (IQR)	16.08 (8.15–40.17)	36.39 (22.52–64.19)	< 0.001^c^
Intraventricular hemorrhage, *n* (%)	20 (36%)	24 (53%)	0.076^a^
Anticoagulants, *n (%)*	6 (11%)	7 (16%)	0.470^a^
rPS, median (IQR)	1.70 (1.27–3.54)	2.36 (1.44–5.90)	0.184^c^
Admission GCS score, median (IQR)	15 (13–15)	11 (8–13.5)	< 0.001^c^

*GCS* Glasgow Coma Scale, *IQR* interquartile range, *rPS* relative blood-brain barrier permeability-surface area product, *SD* standard deviation

^a^indicates χ^2^-test

^b^indicates Student’s t-test

^c^indicates Mann Whitney U-test

**Table 4:** Multivariate logistic regression analysis for ICH patients with poor outcome including variables with the prespecified univariate *p* ≤ 0.1

Variable	Odds ratio	95% confidence interval	*p* value
Hematoma volume (mL)	1.054	1.014–1.096	0.008
Admission GCS score	0.846	0.745–0.961	0.010
Perihematomal edema volume (mL)	1.011	0.990–1.032	0.308
Intraventricular hemorrhage	1.515	0.572–4.013	0.404

*GCS* Glasgow Coma Scale

## Discussion

In our study, we investigated the temporal evolution of perihematomal BBB compromise and edema growth after hemorrhage using CTP data collected from patients in different intracerebral hemorrhage phases. We found that perihematomal BBB permeability focally increases in a biphasic pattern, and edema increases gradually over time until the late subacute phase. BBB compromise was associated with edema growth around the hematoma. These results suggested that BBB compromise is a course of dynamic change, and disruption of BBB integrity may influence cerebral edema formation and growth. These findings may help to guide clinical interventions aimed at alleviating BBB compromise and edema growth to improve the prognosis of patients with intracerebral hemorrhage.

We observed dynamic changes in the perihematomal BBB permeability with two increases after intracerebral hemorrhage onset. A few previous studies have paid attention to the presence of high BBB permeability within a certain phase after hemorrhage [14, 15, 20], and most results on this topic are derived from animal studies [16, 17]. In rodent intracerebral hemorrhage models, the only elevation of perihematomal BBB permeability occurred within 1 week following hemorrhage [16, 17]. Different from this result, our study showed two elevations of perihematomal BBB permeability after hemorrhage, and the second elevation over 1 week found in our study has never been reported before. Additionally, in this study, we observed that increased BBB permeability is associated with edema growth after ictus, which indicates that the BBB condition may regulate cerebral edema formation and growth in intracerebral hemorrhage patients.

In a few recent studies post-intracerebral hemorrhage, the elevated level of BBB permeability in the perihematomal region has been observed as early as within 6 h and it has been reported to peak within 1–3 days [16, 21, 22]. Our results indicate that BBB permeability begins to increase within the first 24 h after intracerebral hemorrhage onset and reaches the peak level in the acute phase. This finding is in line with previous experimental intracerebral hemorrhage research [21, 22]. Moreover, our study revealed a significant impact of perihematomal BBB permeability on edema formation in the acute phase after hemorrhage in which edema developed rapidly and some patients showed neurological deterioration [23–25]. Therefore, the first elevation of perihematomal BBB permeability deserves more attention, and it may act as a potential intervention target in alleviating early edema formation and maintaining stable neurological conditions in intracerebral hemorrhage patients.

We also observed a delayed increase in perihematomal BBB permeability in the late subacute and chronic intracerebral hemorrhage phase. Different from our findings, previous experimental intracerebral hemorrhage models have presented a decreasing trend of perihematomal BBB permeability from approximately day 7 to day 11 after intracerebral hemorrhage [16, 21]. In our opinion, the inconsistency of perihematomal BBB condition after 1 week of intracerebral hemorrhage may reflect the differences in cerebral structural characteristics between animal models and humans [26]. The collagenase-induced or blood infusion experimental intracerebral hemorrhage may not perfectly reflect the real pathophysiological state of intracerebral hemorrhage patients. We think that the second increase of perihematomal BBB permeability after hemorrhage may be the consequence of delayed BBB damage by toxic substances such as free iron, which reaches a peak level on day 7 and maintains a high concentration until day 14 after intracerebral hemorrhage [27, 28].

Additionally, the neovascularity of perihematomal brain tissue may also contribute to the delayed increase of perihematomal BBB permeability [29, 30]. Intracerebral hemorrhage-induced damage may lead to the disruption of BBB integrity and resultantly trigger edema formation and growth [18, 19]. Following this, the BBB repair mechanisms may promote functional recovery and structural reconstruction to alleviate edema formation and accelerate edema absorption [31]. The neovascularity of brain tissue plays an important role in the repair of BBB following hemorrhage [31, 32]; however, the development of neovascularity is immature in late subacute and chronic phases after hemorrhage compared to normal vessels and it may lead to the delayed increase of perihematomal BBB permeability [29, 30]. Moreover, we also found a significant relationship between perihematomal BBB permeability and edema volume in the late subacute phase after hemorrhage which is compatible with a dynamic contrast-enhanced magnetic resonance imaging study [20]. These results indicate that perihematomal BBB compromise 1 week following ictus may play a crucial role in delayed vasogenic edema formation and growth and functional outcomes.

Significant correlations between perihematomal BBB permeability and edema volume have been shown in several recent studies [15, 20]. Our study reveals a strong linear association between higher BBB permeability and larger edema volume, especially in acute and late subacute phases after intracerebral hemorrhage. These results further confirm previous clinical studies that demonstrated the important role of BBB compromise in edema formation and growth at these stages [15, 20]. In our study, perihematomal BBB permeability and edema volume, and the relationship between the two changed significantly in the course of intracerebral hemorrhage. Such an analysis of the relationship between the perihematomal BBB permeability and edema volume has not been done before and hence it may reveal novel insights. In this study, BBB condition was not associated with edema volume in the hyperacute phase after intracerebral hemorrhage onset. In this phase, the perihematomal edema formation may have been dominated by the exudative brain edema promoted by clot retraction and cytotoxic effect, with the state of BBB compromise around hematoma not having a significant influence [13, 33]. This thinking also explains the results of a clinical study that indicated no association between BBB condition around hematoma with edema volume at baseline or subsequent periods [14].

Compared with the hyperacute phase, our results showed obvious increases in perihematomal BBB permeability and greater edema growth rates in acute and late subacute phases. These findings are consistent with several previous studies that revealed the continuous growth of perihematomal edema within 2 weeks after ictus [24, 34]. A few studies have also indicated a growth in edema even after 2 weeks following hemorrhage [35]. Therefore, even in the late subacute phase, the important role of perihematomal BBB compromise and edema growth in the cascade of secondary damage of perihematomal tissue after intracerebral hemorrhage needs special attention.

In our study, larger hematomal volumes were observed to more likely present with higher perihematomal BBB permeability. These observations are consistent with the literature. Several related studies demonstrated that perihematomal BBB permeability was found to be higher in patients with larger hematomal volumes than those with small hematomal volumes in acute and late subacute phases after hemorrhage [15, 20]. Our results demonstrated that there is no significant difference in perihematomal BBB permeability between lobar and deep hemorrhages, which seems to be contradictory to a previous study [20]. A reasonable explanation for the inconsistent result may be that they reflect differences in study design and methodology, inclusion criteria, as well as differences in etiologies of intracerebral hemorrhage. The related research indicated that lobar hemorrhages are often secondary to amyloid angiopathy or coagulopathy, and hemorrhages in deep locations are usually derived from hypertension [20]. In our study, the majority of intracerebral hemorrhage patients were attributed to hypertension both for lobar and deep hemorrhages. Therefore, it is reasonable to speculate that the difference in perihematomal BBB permeability between lobar and deep hemorrhages may be due to the effect of different etiologies but not hemorrhagic locations.

Hematoma size and perihematomal edema growth are generally regarded to be the strongest two predictors of mortality and outcome following spontaneous intracerebral hemorrhage [4, 36]. We confirmed that hematoma volume and admission GCS score are independent factors for poor prognosis at 90 days after hemorrhage, and the results are consistent with the literature [36, 37]. The significant association between perihematomal BBB permeability and mRS score at 90 days in post-intracerebral hemorrhage patients was not observed in our study. This result seemed to be in conflict with a previous study using diethylenetriamine pentaacetic acid single-photon emission computer tomography which showed that BBB permeability measured at 24–48 h after hemorrhage was associated with functional outcomes at 3 and 6 months [38]. In our opinion, this discordance may be due to the small proportion of intracerebral hemorrhage patients who underwent BBB integrity assessment during this time interval and the limited sample in the single phase of our study.

There is evidence to suggest that in the progression of intracerebral hemorrhage, many mechanisms are involved in perihematomal BBB compromise and edema rapid growth after intracerebral hemorrhage onset [38]. Existing evidence from animal studies suggests that multiple injury factors, such as thrombin release, hemoglobin degradation, erythrocyte lysis, matrix metalloproteinase‑9 increase, vascular endothelial growth factor upgrade and dynamic changes in cerebral blood flow, etc. contribute to perihematomal BBB disruption and edema formation [39–41]. The original intention of our study was to elucidate the temporal evolution of BBB comprise and relationship between BBB disruption and edema growth, so this study is not equipped to evaluate the effect of perihematomal BBB injury that may come from these or other mechanisms. Future studies should further clarify how perihematomal BBB compromise is related to edema formation and growth, and whether BBB permeability may predict functional outcome as an imaging marker in patients with intracerebral hemorrhage.

This study has a few limitations. Firstly, rather than longitudinal assessments, we performed only a single measurement of perihematomal BBB permeability. Although sequential measurements at multiple time points would have been ideal in the evolution of BBB disruption following intracerebral hemorrhage in humans, in clinical settings, it is relatively infeasible to undergo multiple CTP examinations. Secondly, the use of CT as initial admission imaging, rather than magnetic resonance imaging, also imposed limitations on the accuracy of hematoma and perihematomal edema volume measurements. Additionally, the differences between edema and normal brain tissue are subtle on CT images, and identification of edema boundaries may be susceptible to observer error. Thirdly, multiple isolated ROIs measurements in the region around hematoma may lead to smaller measuring fields compared with covering entire perihematomal rim but this measurement may avoid the influence of major blood vessels and other undesirable structures. Fourthly, CTP measurement technique used in our study was first-pass acquisition, and first-pass CTP estimates of BBB permeability tend to overestimate contrast leakage rates. Finally, our cohort of 101 patients is not a sizeable sample that limits the confidence with which we can confirm an interaction between perihematomal BBB compromise and edema growth. One of strengths of our study is that we for the first time characterized the temporal evolution of perihematomal BBB compromise and edema growth in intracerebral hemorrhage patients by collecting a large amount of data at different phases after hemorrhage.

In summary, in this study we observed a pattern of dynamic changes in BBB permeability around the hematoma in intracerebral hemorrhage patients. Perihematomal edema growth presented a continuous upward trend until late subacute phase after hemorrhage. Higher BBB permeability was associated with larger perihematomal edema volumes. These findings suggest that BBB compromise is likely to be an important factor in edema growth, especially in acute and subacute phases after intracerebral hemorrhage. Further studies are needed to assess the impact of BBB compromise on intracerebral hemorrhage patient outcomes. Quantitative assessment of BBB compromise after hemorrhage may reflect the severity of secondary brain injury, and guide future therapeutic interventions to maximize potential intracerebral hemorrhage patient recovery.

## Supplementary Information

The results of validation analysis after excluding patients with anticoagulants therapy history.
