# Supplementary material for: Temporal Evolution of Perihematomal Blood-Brain Barrier Compromise and Edema Growth After Intracerebral Hemorrhage
Source: Clin Neuroradiol. 2023 Apr 25;33(3):813–24. doi: 10.1007/s00062-023-01285-z (PMC10449681; doi:10.1007/s00062-023-01285-z)
Supplement: Supplementary file 1 — The results of validation analysis after excluding patients with anticoagulants therapy history. [file 62_2023_1285_MOESM1_ESM.docx]

**Supplementary Materials**

**1.Temporal evolution of perihematomal blood-brain barrier (BBB) permeability and edema growth**

Of the 88 included patients without medication history of anticoagulants, the characteristics are summarized in Supplementary Table 1 according to different phases. BBB permeability showed an early increase in the hyperacute and acute phase, a decrease in the early subacute phase, and a delayed increase in the late subacute and chronic phases (Supplementary Fig. 1A). The perihematomal edema volume gradually increased over time within the first 2 weeks and slightly increased thereafter (Supplementary Fig. 1B).

**2.Relationship between perihematomal relative BBB permeability-surface area product (rPS) and edema with time**

In 88 included patients, univariate linear regression analysis showed that the rPS value in the perihematomal region was significantly associated with perihematomal edema volume (β = 0.389; *p* < 0.001). The association remained significant in the multivariate regression analysis that included hematoma volume as a covariate (β = 0.272; *p* = 0.006) (Supplementary Fig. 2A). In the sub-group analysis, rPS value in acute phase was associated with perihematomal edema volume (β = 0.689; *p* < 0.001), even after including hematoma volume as a covariate (β = 0.487; *p* = 0.012) (Supplementary Fig. 2B). Similar relationship was observed in the late subacute phase (univariate analysis: β = 0.623, *p* = 0.003), but it did not remain significant in multivariate analysis when including hematoma volume as a covariate (β = 0.297; *p* = 0.079) (Supplementary Fig. 2C).

**Relationship between rPS and other demographic factors**

In 88 included patients, higher rPS values were significantly related to larger hematoma volume in all patients (β = 0.308, *p* = 0.003) (Supplementary Fig. 3A) and patients with deep hemorrhage (β = 0.326, *p* = 0.006) (Supplementary Fig. 3B), but not in patients with lobar hemorrhage (β = 0.271, *p* = 0.277) (Supplementary Fig. 3C). There is no relationship between perihematomal rPS value and age (β = 0.109, *p* = 0.310). Mann-Whitney U test revealed no significant difference in perihematomal rPS value between ICH patients with deep and lobar hemorrhages (z = − 1.055, *p* = 0.291), female and male patients (z = 0.588, *p* = 0.556), and with and without prior hypertension (z = 1.271, *p* = 0.204) and diabetes (z = − 0.304, *p* = 0.761).

**Supplementary Table 1** Characteristics in ICH patients grouped by time interval from ICH onset to CTP examination

| Characteristics | Hyperacute (<1d)  n=21 | Acute (1-3d)  n=20 | Early subacute (3-7d)  n=22 | Late subacute (7-14d)  n=21 | Chronic (>14d)  n=4 |
| --- | --- | --- | --- | --- | --- |
| Age, mean ± SD (y) | 46.14 ± 14.67 | 53.60 ± 8.08 | 49.82 ± 11.03 | 52.57 ± 11.93 | 52.25 ± 9.07 |
| Sex (female), n (%) | 7 (33%) | 8 (40%) | 5 (23%) | 5 (24%) | 2 (50%) |
| Admission GCS, median (IQR) | 13 (9-15) | 12 (8-15) | 12.5 (5.75-15) | 14 (12.5-15) | 15 (14.25-15) |
| Admission NIHSS, median (IQR) | 11 (2.5-16) | 9.5 (6-16.5) | 7 (3.75-9.5) | 6 (1-10) | 7.5 (4-12.5) |
| Hematoma volume (mL), median (IQR) | 25.5 (8.74-39.02) | 15.91 (5.73-33.16) | 11.66 (5.00-18.04) | 5.97 (2.50-13.41) | 6.03 (5.03-13.97) |
| Perihematomal edema volume (mL),  median (IQR) | 16.76 (9.26-38.98) | 24.78 (15.13-55.48) | 26.52 (11.01-50.94) | 22.51 (10.56-46.56) | 39.28 (12.73-92.32) |
| Relative perihematomal edema volume (mL),  median (IQR) | 1.09 (0.55-2.01) | 1.85 (1.06-2.91) | 2.41 (1.78-4.18) | 3.66 (1.86-6.20) | 3.68 (2.13-14.42) |
| Hematoma location (deep), n (%) | 16 (76%) | 16 (80%) | 17 (77%) | 19 (81%) | 4 (100%) |
| rPS (all), median (IQR) | 1.53 (1.28-2.40) | 2.77 (1.60-5.39) | 1.33 (1.12-2.07) | 2.83 (1.57-8.85) | 2.37 (1.81-15.14) |
| rPS (deep), median (IQR) | 1.79 (1.27-2.48) | 2.78 (1.63-7.00) | 1.31 (1.11-2.83) | 2.83 (1.63-6.75) | 2.37 (1.81-15.14) |
| rPS (lobar), median (IQR) | 1.36 (1.19-5.31) | - | 1.34 (1.18-1.72) | - | − |

*GCS* Glasgow Coma Scale, *IQR* interquartile range, *NIHSS* National Institutes of Health Stroke Scale, *rPS* relative blood-brain barrier permeability-surface

area product, *SD* standard deviation

**
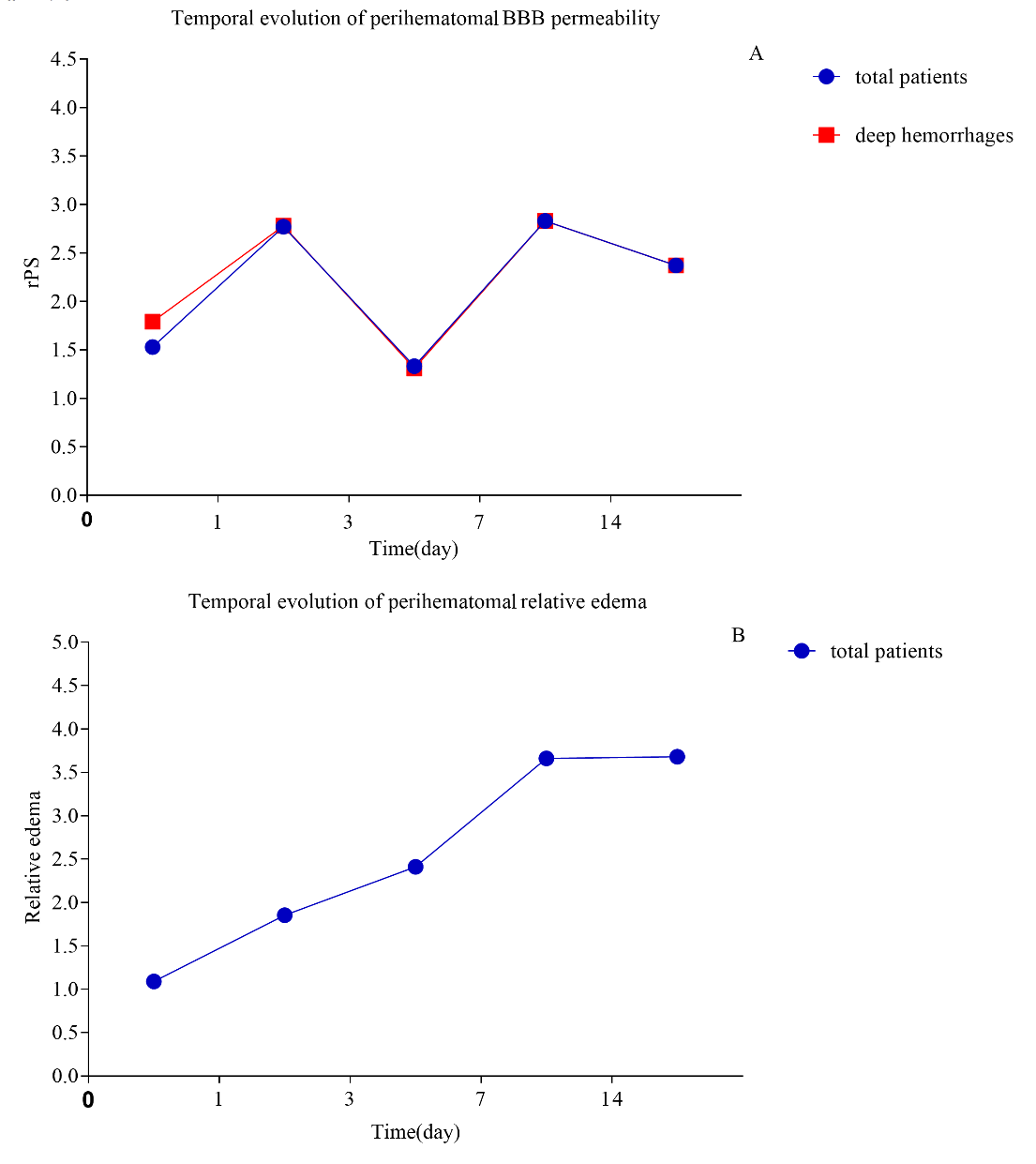
**

**Supplementary Figure 1**

Temporal evolution of perihematomal BBB (A) and relative perihematomal edema(B) in intracerebral hemorrhage patients without medication history of anticoagulants. *BBB* blood-brain barrier, *rPS* relative BBB permeability-surface area product

**
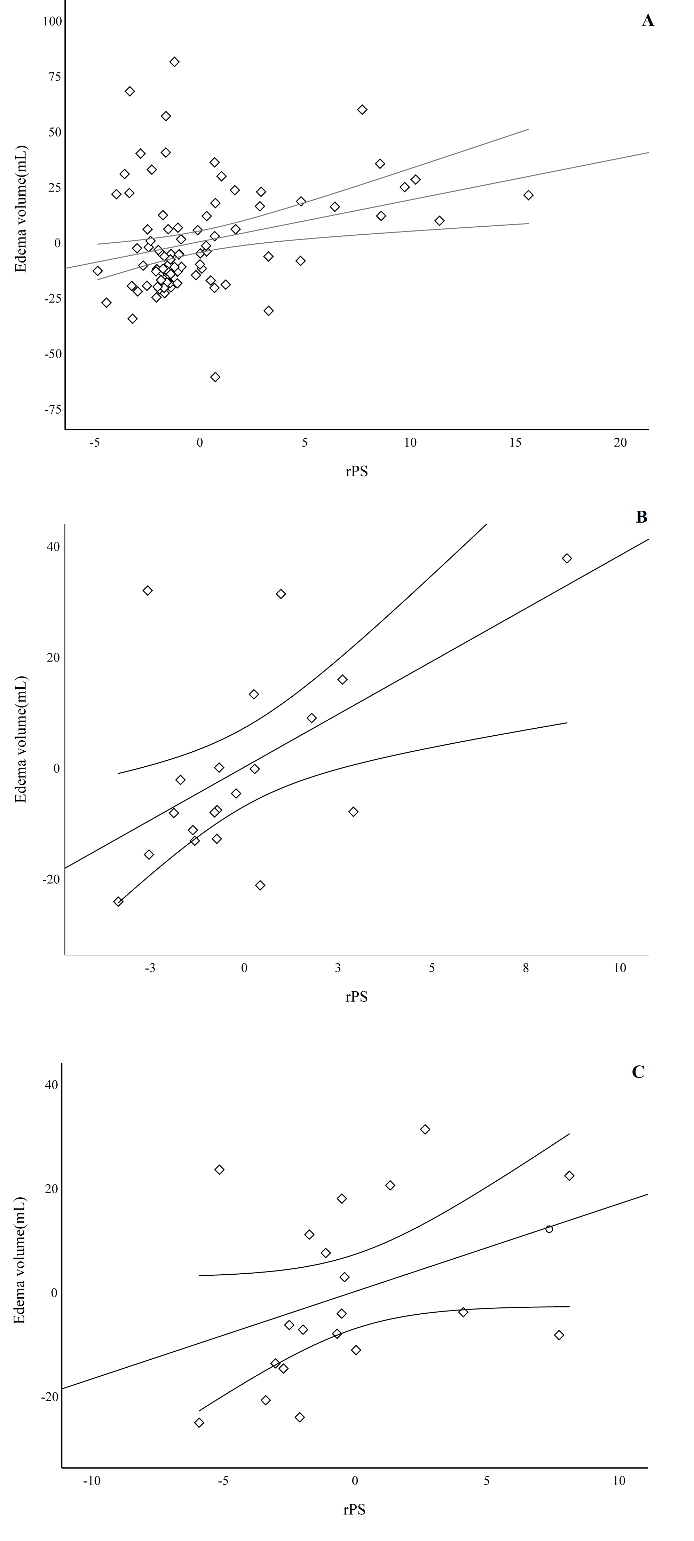
**

**Supplementary Figure 2**

Partial Regression plots showing the relationships between blood-brain barrier permeability and perihematomal edema volume in all patients without medication history of anticoagulants(A), patients in acute phase(B), patients in late subacute phase (C). rPS is associated positively with the perihematomal edema volume in all patients without medication history of anticoagulants, patients in acute when using hematoma volume as a covariate. A similar trend could be found in patients in late subacute phase. *rPS* relative blood-brain barrier permeability-surface area product

**
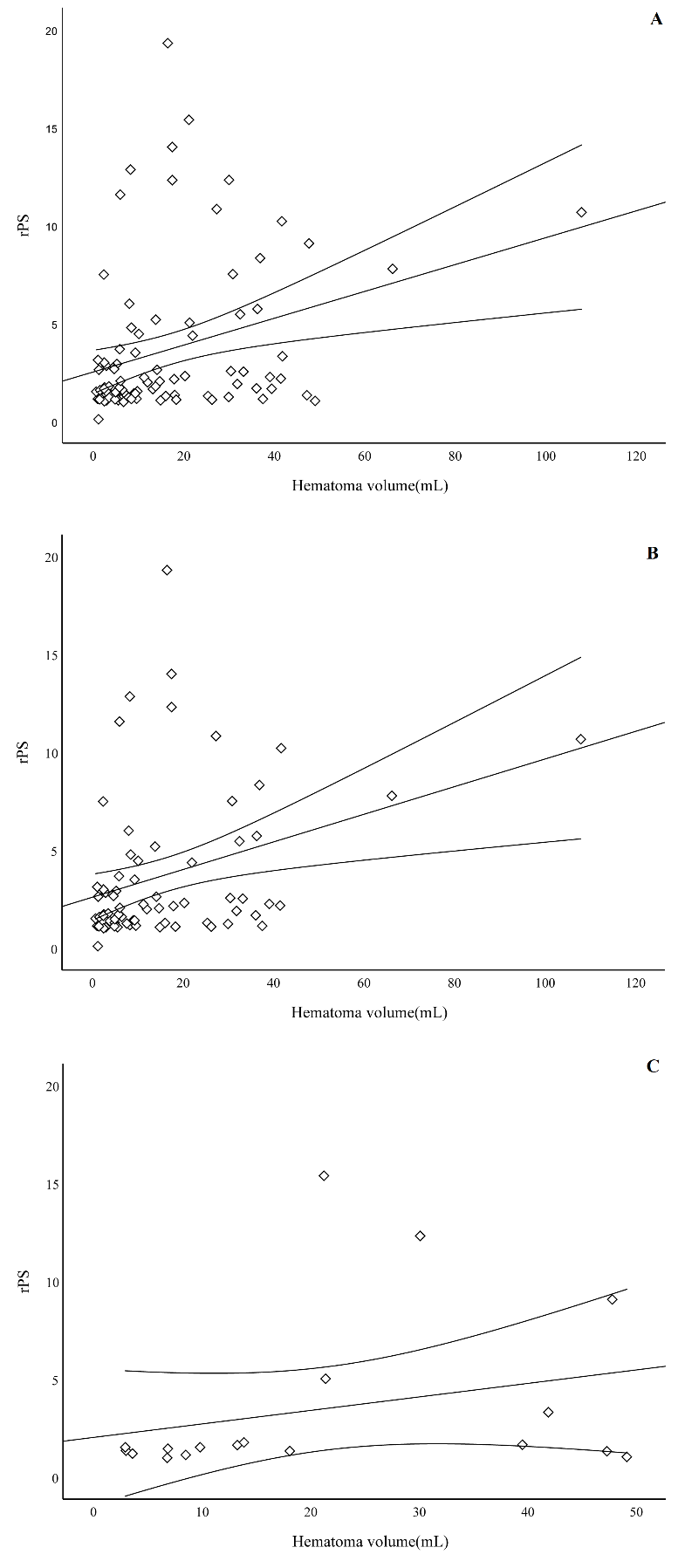
**

**Supplementary Figure 3**

Scatter plots with linear regression coefficients and 95% confidence intervals. Perihematomal rPS is associated positively with the hematoma volume in all patients without medication history of anticoagulants(A) and patients with deep hemorrhage (B), but not in lobar hemorrhage patients (C). *rPS* relative blood-brain barrier permeability-surface area product.
